# Breeding behavior in the blind Mexican cavefish and its river-dwelling conspecific

**DOI:** 10.1371/journal.pone.0212591

**Published:** 2019-02-20

**Authors:** Victor Simon, Carole Hyacinthe, Sylvie Rétaux

**Affiliations:** Paris-Saclay Institute of Neuroscience, CNRS UMR9197, Université Paris-Sud, Université Paris-Saclay, Gif-sur-Yvette, France; University of Cincinnati, UNITED STATES

## Abstract

Fish reproductive patterns are very diverse in terms of breeding frequency, mating system, sexual dimorphisms and selection, mate choice, spawning site choice, courtship patterns, spawning behaviors and parental care. Here we have compared the breeding behavior of the surface-dwelling and cave-dwelling morphs of the characiform *A*. *mexicanus*, with the goals of documenting the spawning behavior in this emerging model organism, its possible evolution after cave colonization, and the sensory modalities involved. Using infrared video recordings, we showed that cave and surface *Astyanax* spawning behavior is identical, occurs in the dark, and can be divided into 5 rapid phases repeated many times, about once *per* minute, during spawning sessions which last about one hour and involve one female and several males. Such features may constitute “pre-adaptive traits” which have facilitated fish survival after cave colonization, and may also explain how the two morphs can hybridize in the wild and in the laboratory. Accordingly, cross-breeding experiments involving females of one morphotype and males of the other morphotype showed the same behavior including the same five phases. However, breeding between cavefish females and surface fish males was more frequent than the reverse. Finally, cavefish female pheromonal solution was able to trigger strong behavioral responses in cavefish males–but not on surface fish males. Lastly, egg production seemed higher in surface fish females than in cavefish females. These results are discussed with regards to the sensory modalities involved in triggering reproductive behavior in the two morphs, as well as its possible ongoing evolution.

## Introduction

The characiform fish *Astyanax mexicanus*, or Mexican tetra, comes in two distinct forms. The river-dwelling morphs inhabit South, Central America and Texas, and the blind depigmented cave-dwelling morphs live in the permanent darkness of 30 caves in North-Eastern Mexico [1, 2]. Several lines of evidence (genetic, phylogeographic, developmental) indicate that the two forms derive from a common ancestor that was “surface fish–like” and was eyed and pigmented (see reviews in [3]). Importantly, cave colonization occurred recently, probably less than 25.000 years ago [4, 5], suggesting that cave adaptation was rapid. This system is unique because it gives the opportunity to compare extant surface fish and several cavefish populations of the same species, and genetic analyses involving the production of hybrids are possible. The two forms of *A*. *mexicanus* have thus become popular for evolutionary biology studies focusing on the mechanisms of adaptation after a drastic environmental change, and on the analysis of troglomorphic features.

Surface and cave *A*. *mexicanus* differ by many traits in terms of morphology, physiology and behavior, yet they belong to the same species, with reproductive isolation and interfertility taken as criteria to define a species. Indeed, cave and surface morphs can reproduce and give a fertile progeny. This holds true both in the laboratory where crosses between morphs is the basis for genetic studies [6–10], and in the wild where hybridization can occur after surface fish are washed into caves after flooding during the rainy season [2, 11]. It posits that there is no barrier to reproduction between the two morphs in terms of sex determination mechanism and behavior. However, there is an important lack of knowledge on *A*. *mexicanus* reproductive behavior, on its possible evolution after cave colonization, and on the sensory modalities that help or trigger this behavior. Moreover, the breeding behavior of cavefishes in general remains enigmatic, even in the long-studied Amblyopsid cavefishes of North America [12].

Fish reproductive patterns are very diverse in terms of breeding frequency, mating system, sexual dimorphisms and selection, mate choice, spawning site choice, courtship patterns, spawning behaviors and parental care [13]. In the lab, *A*. *mexicanus* can repetitively produce thousands of eggs every other week all along the year [14, 15], but whether there is seasonality of reproduction in the wild is unknown. Small characids of the genus *Astyanax* have extended spawning seasons that can last from spring to autumn [16]. The presence of fry has been reported in the Pachón cave both during the rainy and the dry season, which may suggest that breeding can occur all year long in this cave which does not receive any flooding [17]. This seems different from the case of Amblyopsid cavefish, a family of freshwater fishes found in the southern and eastern United States, in which reproduction occurs after spring floods, when food is abundant [12]. A. *mexicanus* in the lab are promiscuous breeders, with little or no choice of males and females reproducing with multiple partners -although a preference for large males has been reported for surface fish females in the light but not in the dark [18]. Interactions between males and females could be facilitated by the presence of small denticles on the male’s anal fin that likely helps the male hook the spawning female [14, 15]. Concerning the courtship and spawning, a preliminary description was given by Wilkens 45 years ago [19], stating that males become “activated” by females and start swimming rapidly. When encountering a mature female, the two fish swim close together and release sperm and eggs. It was also stated that male “activation” depends on the olfactory system because olfactory nerve transection abolishes the behavior. In this first laboratory study, no major difference was found between surface fish and cavefish originating for the Pachón and Los Sabinos caves [19]. Finally, *A*. *mexicanus* show no parental care and on the contrary, after spawning, both morphs show filial cannibalism and tend to eat their own eggs [15]. The offspring would serve as an alternative food source and improve future reproductive success.

Reproductive behavior is key to the perpetuation of the species on one hand, and to the process of speciation on the other hand, two processes that are highly relevant to the case of cavefishes living and evolving in a very special environment. In the rapidly-evolving cichlid species of the great African lakes, the divergence of reproductive color signals (coloration in males and visual perception in females) causes reproductive isolation and leads to speciation [20]. Divergent sexual selection acting on other sensory modalities, including acoustic [21, 22] and perhaps also chemical communication produced during courtship may also intervene in speciation.

*Astyanax mexicanus* has become an important fish model for evolution studies, and a thorough qualitative and quantitative description of courtship and spawning behavior is lacking for this species, and is necessary to discuss its ongoing adaptive evolution in caves and proposed ongoing ecological speciation [23]. Here, we document in detail the reproductive behavior of surface fish and Pachón cavefish, in laboratory conditions. We find that the two morphs show exact identical behaviors, including during inter-morph cross-breeding. We also provide evidence that olfactory cues released by females ready to spawn can trigger male reproductive behavior.

## Materials and methods

### *A*. *mexicanus* fish

Laboratory stocks of *A*. *mexicanus* surface fish (originating from San Solomon Spring, Balmorhea State Park, Texas, USA) and cavefish (originating from the Pachón cave, San Luis Potosi, Mexico) were obtained in 2004 from the Jeffery laboratory at the University of Maryland, College Park, MD. Since then, in our facility, fish were bred and maintained at 23° C (cavefish) or 26°C (surface fish) on a 12:12 hours light:dark cycle [15]. Animals were treated according to the French and European regulations for handling of animals in research. SR’s authorization for use of animals in research including *Astyanax mexicanus* is 91–116. The Paris Centre-Sud Ethic Committee approved the protocol authorization number 2017–04#8545 related to the present research. Anesthesia, euthanasia, or any kind of animal sacrifice were not part of the study.

### Recording of breeding behavior

Six adult fish aged between 3 and 4 years old (4 males, 2 females) were transferred into a 34 liter tank (L : 50cm, W : 25cm, H : 30cm) in a temperature- and noise-controlled behavior room. The tank contained clean tap water, a Eheim aquaball 130 filter, an Eheim airpump400, a 50W Eheim Jager heater to control the water temperature at 22°C, and pouzzolane lava rock. Fish were habituated in this experimental tank during 24 hours, then the temperature was raised to 26°C to induce spawning.

**Fig 1:**
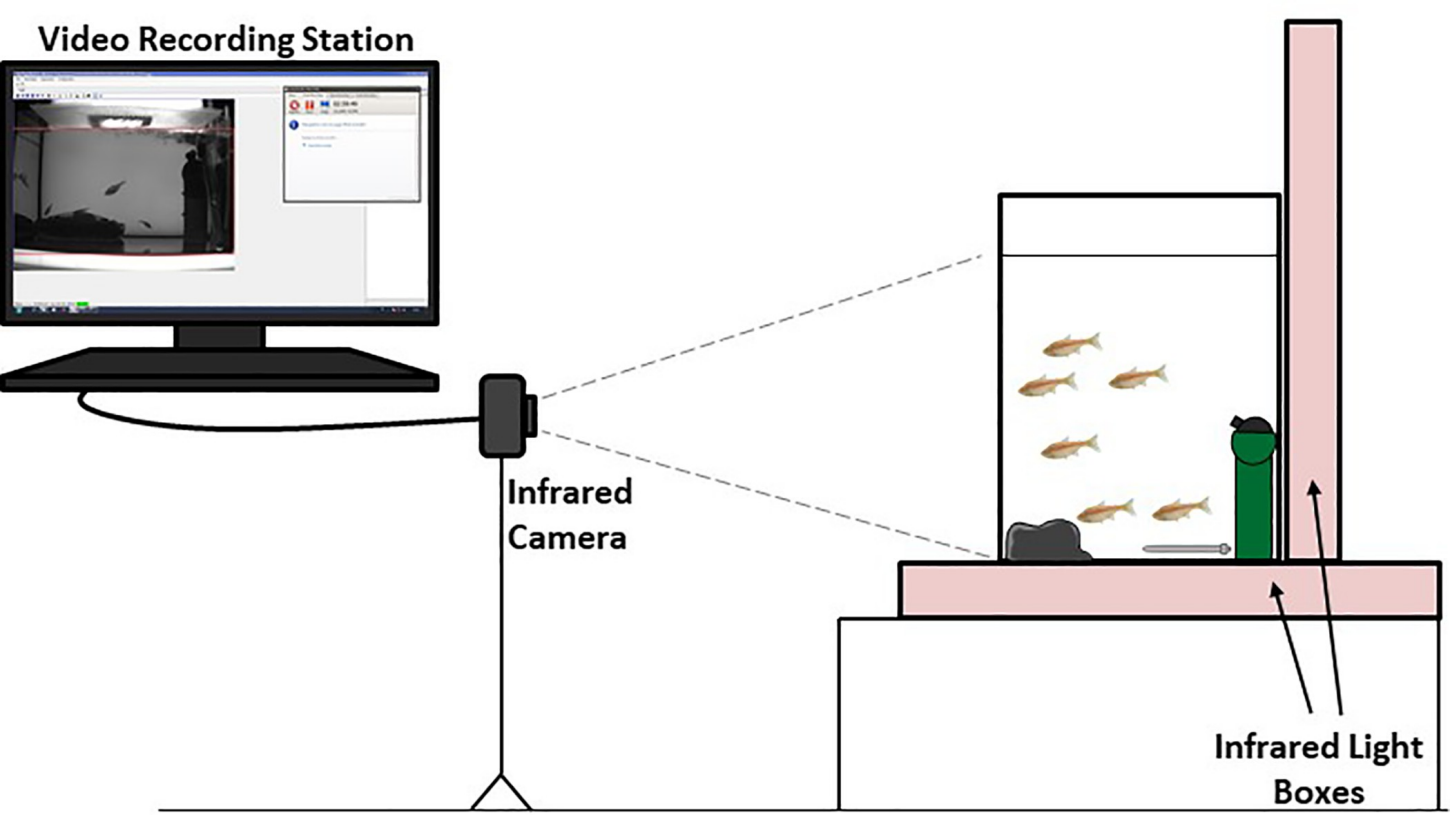
Recording setup. Infrared light boxes were placed at the back and bottom of a tank containing 6 fish, an oxygenating pump (green), a heating resistance (grey) and pouzzolane in a net (black). Recordings were obtained overnight with an infrared camera. Drawing not to scale.

For the purpose of night-time infrared video recordings, two infrared light sources (Viewpoint) were placed under and behind the tank, and a Dragonfly2 camera (Point Grey) was placed in front of the tank and connected to a Viewpoint imaging software (Fig 1). Three different groups of surface fish and 3 different groups of cavefish were recorded, each during 4 consecutive nights. Of these, a total of 3 breeding nights were obtained for surface fish, and 5 breeding nights were obtained from cavefish. For the cross-breeding experiments, 3 groups of female cavefish/male surface fish and 5 groups of female surface fish/male cavefish were recorded.

### Analysis of breeding behavior

For analysis, videos were manually/visually inspected, interpreted and scored. All results are presented *per* breeding session for one female. Statistical comparisons were performed using non-parametric Mann-Whitney tests.

For the estimation of the number of eggs spawned *per* female and *per* spawning period, eggs were counted in a few spawning events where expulsion was well contrasted on the movie and clutch size could be accurately estimated (n = 8 for cavefish; n = 10 for surface fish). The total quantity of eggs released by female was then estimated by multiplying this number by the number of time egg spawning was observed during a spawning session.

Statistical analyses were performed under GraphPad Prism8, using non parametric Mann Whitney tests.

### Pheromone assays

Four adult males aged between 3 and 4 years old were transferred into a 20 liter tank (L : 40cm, W : 20cm, H : 25cm) in a temperature- and noise-controlled behavior room, with the same infrared recording set-up as described above. Perfusion was made by opening the Luer stopper of a medical solution administration tubing (Baxter, U.K.) to perfuse solutions at 5mL/min from a syringe reservoir. Video recording was started when the perfusion of control water solution was initiated, during 8 minutes. This served as a control for possible attraction of the fish to the water flow. This was followed by perfusion of a total of 40 ml of pheromone solution (or water) during 8 additional minutes.

Cavefish female pheromones were obtained as follows. An excited female (identified because of obvious male chasing behavior, see Results section) was fished from a breeding tank in the main facility room (containing ~20–30 fish with a 2 males for 1 female ratio) and was squeezed in order to ascertain that she was ready to spawn, with mature gametes. The female was immediately placed back in the tank of origin, where she soon attracted again the interest of males. The same female was netted again and carefully placed (wearing gloves) into a beaker containing 150 ml of water, in which she was allowed to swim for 15–20 seconds. The female was then transferred back to the tank of origin.

We attempted to collect surface fish female pheromone with the same protocol, but no biological activity could be recovered. It is worth mentioning that surface fish female did not attract so much the interest of males when they were put back in the tank after squeezing. One hypothesis is that the female may be too stressed, rendering difficult the interpretation of the results. Of note, for the interpretation of the absence of quivering response to the cavefish pheromone by surface fish males, the cavefish pheromone solution was systematically tested on cavefish males, and was always found to be active on them. We also observed that the cavefish pheromone solution could be kept at least 48h at 4°C without losing its biological activity.

## Results

### Characterization of reproductive behavior in *A*. *mexicanus* surface fish and cavefish

When maintained on a 12/12-light/dark cycle in the laboratory, both *Astyanax* morphs lay eggs during the night when stimulated by temperature and water changes and provided with a spawning substrate [14, 15]. We therefore designed an experimental setup where the natural breeding behavior could be recorded, without any external perturbation, in the dark (Fig 1). Six fish (2 females + 4 males) were placed in a tank, and infrared videos were recorded during the following 4 consecutive nights. All fish (total n = 36) were aged between 3 and 4 years, and their standard length ranged between 3.9 (smallest male) and 9 cm (largest female).

Three different groups of surface fish and 3 different groups of cavefish yielded 288 hours of video recordings, which were visually inspected. All groups (3 out of 3 surface fish groups; 3 out of 3 cavefish groups) showed reproductive behavior during at least one of the recorded nights, involving the spawning of almost all the females (6/6 surface fish females spawned; 5/6 cavefish females spawned–of note, the females could be individually recognized on the movies by their shape and size). Globally, this shows that reproductive behavior is common and widespread in *A*. *mexicanus*, at least in healthy, well-fed and young adult individuals.

**Fig 2:**
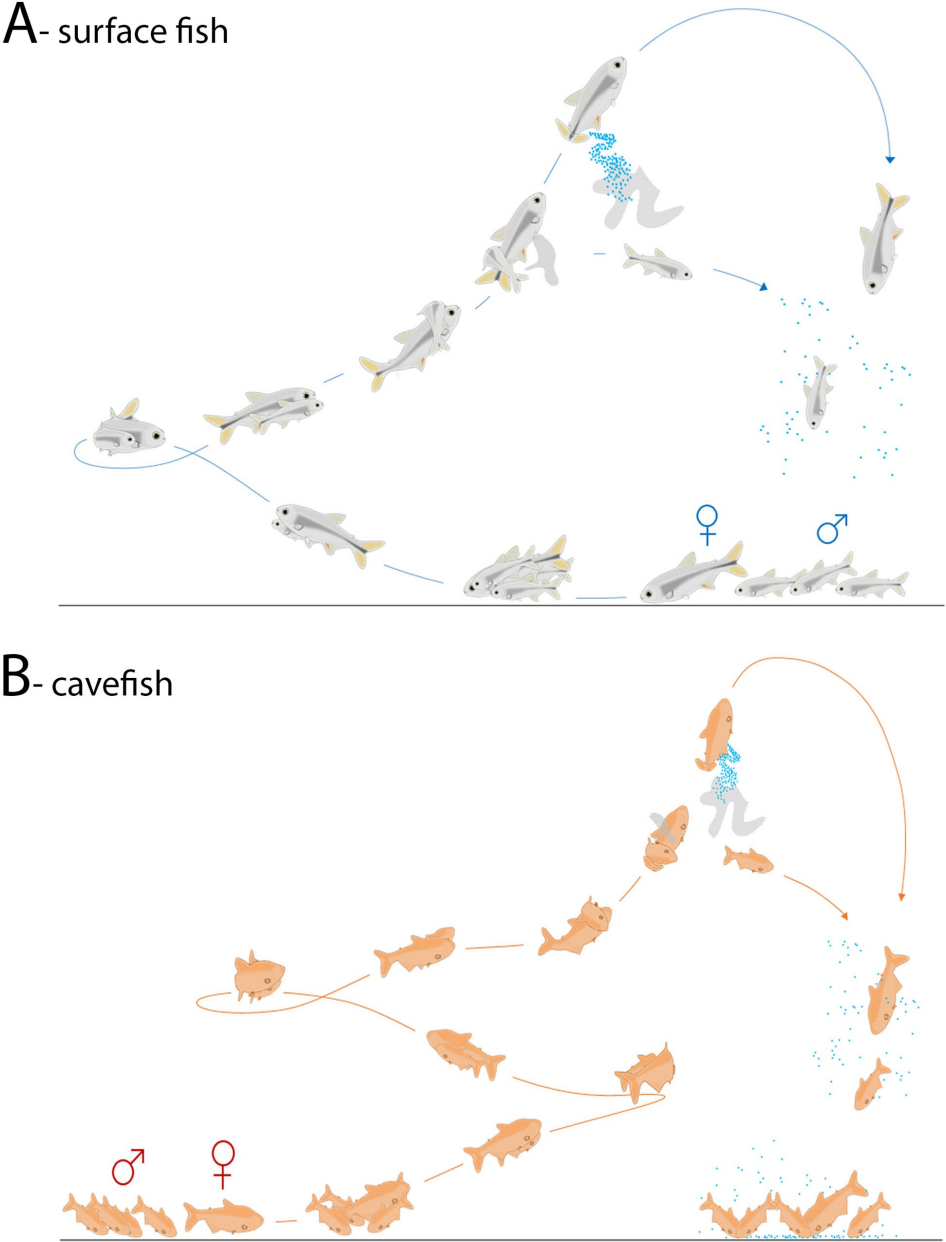
Description of spawning behavior in *A*. *mexicanus*. A (surface fish) and B (cavefish) show realistic drawings capturing the distinct phases of *A*. *mexicanus* behavior in the dark, after careful observation of many spawning events. See also S1 and S2 Movies. The swimming movements and body postures of the male and females are identical in surface fish and cavefish.

Detailed, slow motion observations of the movies allowed to distinctly describe the different phases of the breeding behavior, which were found identical in the two morphs (Fig 2; S1 and S2 Movies). The spawning periods were always preceded (and could be predicted by) an increase in locomotor activity of the female ready to spawn, followed by an increased attraction of the males towards this excited female. The courtship, or reproductive behavior itself could be divided into 5 phases or steps. 1) Bottom swimming of the female. Such locomotion on a two-dimensional plane (instead of 3D in the water column) probably increases the chances of encounter with a male. 2) Chasing by the males. 3) Quiver swimming, or rapid synchronized swimming side by side of the female with one male. 4) Upward swimming and wrapping phase, whereby the female orients and swims towards the surface of the water while the male swims around the female’s back, enwrapping her with a strong curvature of his body, and releasing the sperm. 5) Twitching and egg-laying through a sudden burst of energy and contraction of the female in an upward direction. The expulsed eggs fall down passing through the cloud of sperm, being fertilized. This constitutes the end of the breeding behavior, which lasts 2–3 seconds in total, and the male and the female will then swim distantly and individually again. The whole sequence of 5 phases is repeated many times (see below), involving the same excited female and any of the 4 males present in the tank, constituting a spawning period. Importantly, the viewing of several hundreds of spawning events showed that swimming movements and body postures of the males and the females are identical in surface fish and cavefish (Fig 2). Most often, the two females, and mostly the female that did not spawn, eat the eggs fallen on the bottom of the tank after a spawning bout. The males also feed on eggs at the end of the spawning period, when they do not chase the female anymore.

### Quantification and comparison of reproductive behavior in *A*. *mexicanus* surface fish and cavefish

As shown above, qualitatively and in terms of stereotyped behavior the reproductive manners of the two morphs of *A*. *mexicanus* are identical. We next aimed to quantitatively assess this behavior (Table 1).

**Table 1:** Quantitative aspects of reproductive behavior in *A*. *mexicanus* surface fish and cavefish.

per spawning period, per female	Surface fish (n = 6)	Cavefish (n = 7)	Mann Whitney test
			
Number of spawning attempts	83±29	62±25	NS
Number of complete spawning cycles	61±21	30±17	P = 0.014, U = 4
% of success in accomplishing a spawning cycle	74±6	45±13	P = 0.002; U = 1
			
Total duration of spawning period (min)	77±12	66±28	NS
Number of spawning attempts per minute	0.90±0.34	1±0.44	NS
			
Number of egg-laying events	45±12	17±12	P = 0.004; U = 0
% of success in egg-laying among all spawning attempts	56±11	29±13	P = 0.006; U = 1
Estimated number of spawned eggs	3589±1163	1442±633	P = 0.00042; U = 0
Female standard length (cm)	7.92±0.71	7.40±0.53	NS
Number of eggs per cm of female	436±117	163±108	p = 0.0043, U = 0
			

Values are given as mean ± SD. P and U values are indicated after Mann Whitney tests.

**Fig 3:**
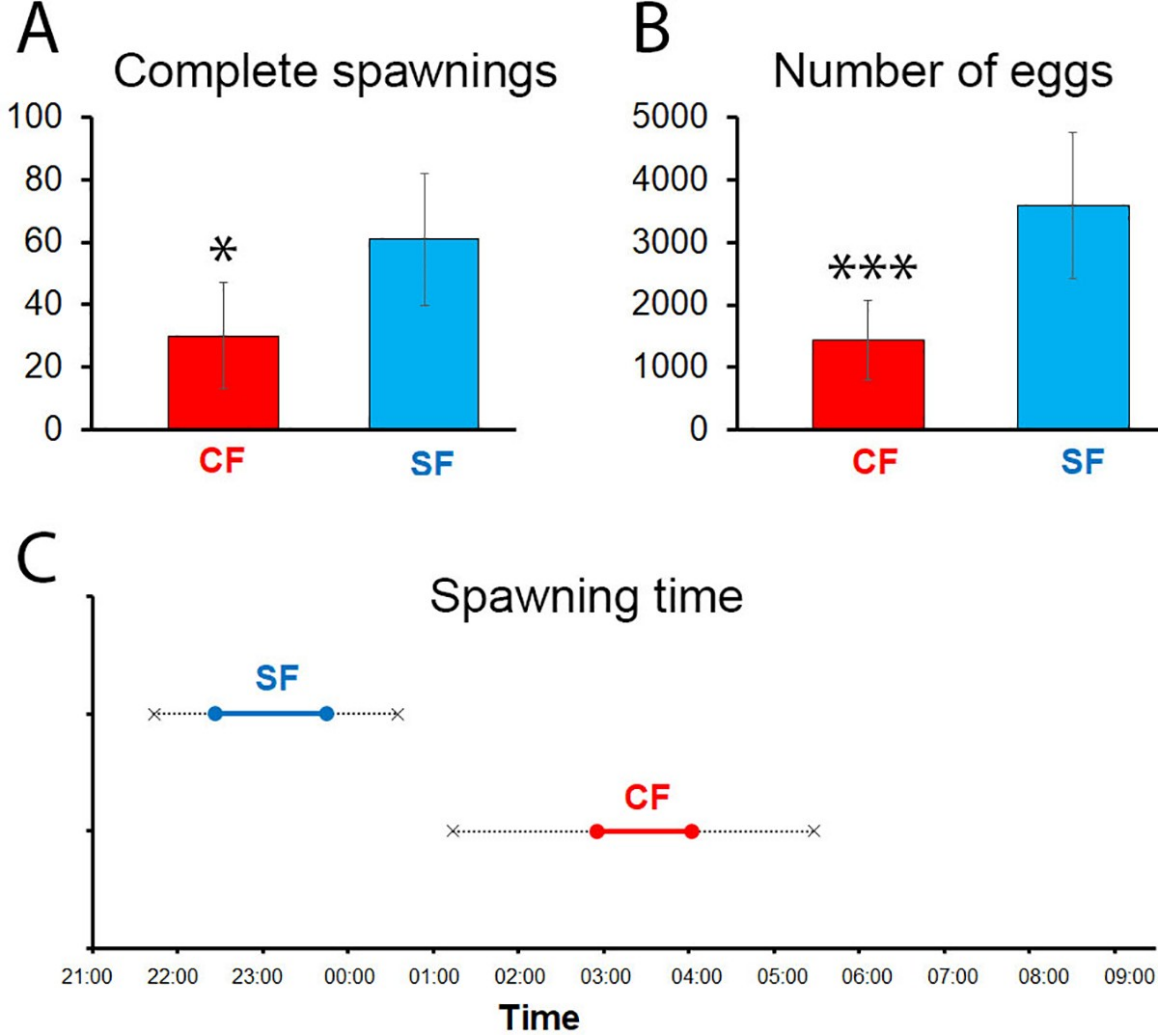
Quantification of spawning behavior, *per* spawning period and *per* female, in *A*. *mexicanus*. A, comparison of the number of completed spawning sequences in surface fish females (SF, blue; n = 6) and cavefish females (CF, red; n = 7) during a spawning period. Results are presented as mean ± SD. B, comparison of the estimation of the number of eggs produced by surface fish females (SF, blue) and cavefish females (CF, red) during a spawning period. C, comparison of the spawning time during the night in surface fish (SF, blue) and cavefish (CF, red).

First, the number of spawning events, *per* spawning period and for one given female, was compared in surface fish and cavefish. While the total number of successive spawning attempts was similar in the two morphs (mean: 83 in surface fish, 62 in cavefish), the number of complete spawning events (i.e, behavioral cycles including all the phases 1 to 5 described above, regardless of whether eggs were laid or not) seem to be higher in surface fish (Fig 3A and Table 1). The success rate in accomplishing the entire courtship sequence was 74% in surface fish, and 45% in cavefish (p = 0.0023, U = 1, n = 6 or 7; Mann Whitney test). The total duration of the spawning period was similar in the two morphs (Table 1), slightly more than an hour, and the frequency of spawning attempts was also similar, about one *per* minute. Thus, spawning appears like a repetitive and demanding behavior in *A*. *mexicanus*.

The number of spawning events giving rise to egg-laying (well visible on the movies) were also counted. They were significantly more numerous for surface fish females than for cavefish females in our conditions (Table 1). Hence, the percentage of successful sequences, ending with egg release, was about twice higher in surface fish than in cavefish (56% versus 29% of the spawning attempts, p = 0.006, U = 1, Mann Whitney test). The number of eggs expulsed *per* female was also estimated (see Methods). Egg clusters released in one spawn were of similar size in the two morphotypes (surface fish: 77±25 eggs, n = 10; cavefish: 68±29 eggs, n = 8), but as the rate of successful egg-laying events was higher in surface fish, the total number of eggs released by one female during the entire spawning period was much higher for the surface-dwelling morphotype, and estimated around 3500 eggs *per* female (Fig 3B and Table 1). As the size of the females may have an important impact on egg mass, the results were also expressed as estimated number of eggs per centimeter of female standard length (Table 1). With this correction by size, a significant difference persisted between surface fish fecundity (p = 0.0043, U = 0, Mann Whitney test). In sum, in our experimental conditions, the reproductive yield seems to be higher in surface fish than in cavefish.

Finally, the time-course of the spawning periods along the night were compared (Fig 3C). Surface fish spawning sessions occurred around 11:00 PM (i.e., 3 hours after the light was turned off), in a very reproducible and well-timed manner. No reproductive events occurred during the remaining night time. Cavefish on the other hand spawned much later, mostly between 3:00 and 4:00AM, and with a greater variability on the spawning time. Of note, it occurred several times that during a given night, the two females present in the tank reproduced. In such case, their breeding periods were either successive or overlapping.

### Cross-breeding between surface fish and cavefish

**Fig 4:**
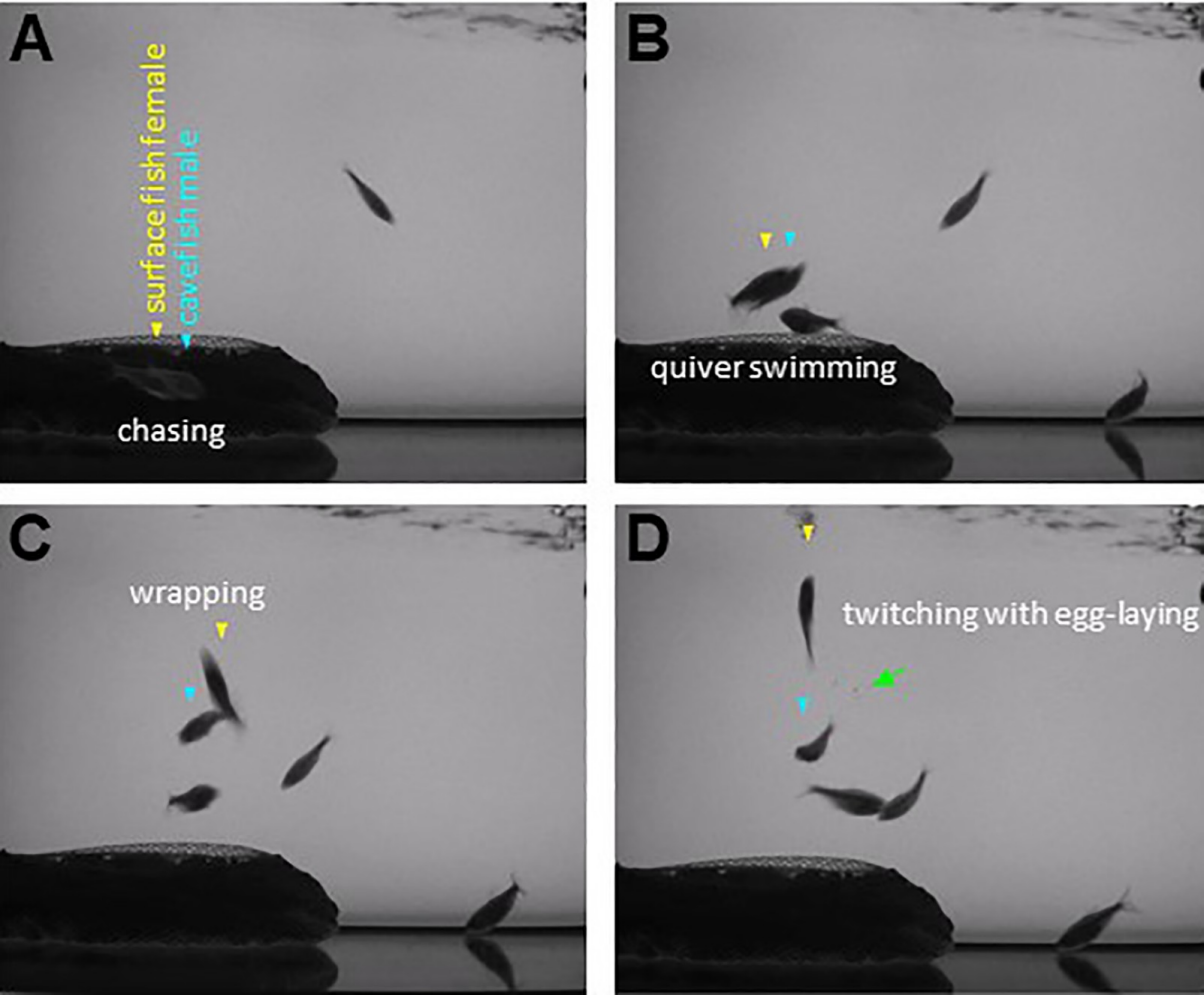
Cross-breeding between surface fish and cavefish. Example of a spawning event between a surface fish female (yellow arrowhead) and a cavefish male (blue arrowhead). Panels A, B, C and D show representative snapshots of the principal phases of the spawning behavior, which ends up with egg release (green arrow) in this case. Note that the other surface fish female (fish in the bottom right corner of the tank in BCD), who is not ready to spawn, does not attract any interest from the males and feeds on the eggs that have been spawned by the active female.

In the wild, hybridization can occur in some caves (e.g., Subterranéo, Chica) when surface fish are washed into the caves after flooding [1, 2, 11]. In the lab as well, F1 hybrids can be obtained through natural spawning. Such phenomenon is probably facilitated by the identical sequence of events constituting the reproductive behavior in surface fish and cavefish (Fig 2), and the spawning behavior occurring during night time, i.e., in the dark for both morphs (Fig 3C) [19]. We thus took advantage of our setup to document the spawning behavior in mixed groups constituted by surface fish females and cavefish males, or *vice versa* (Fig 4; S3 and S4 Movies). The recordings confirm that inter-morph breeding behavior occurs without difficulty when surface fish and cavefish cohabit. Of note, the reproduction between cavefish females and surface fish males was easy to observe and occurred as soon as the first experimental group of 6 fish were put together in a tank. For the reciprocal cross involving surface fish females with cavefish males, a high aggressiveness of the dominant surface fish females was witnessed, rendering courtship behavior difficult to observe. In this case, breeding behavior was observed only after several trials, when the fifth group of 6 fish was constituted.

### Pheromonal trigger of reproductive behavior

Our observations as well as those reported by Wilkens [19] suggested that females which are ready to spawn produce signal(s) that attract the interest of males. In fish, two main sensory components are known to play crucial roles in reproductive behavior: acoustic and chemical communication (see discussion). Here, we tested the involvement of pheromonal communication in *A*. *mexicanus* spawning behavior.

**Fig 5:**
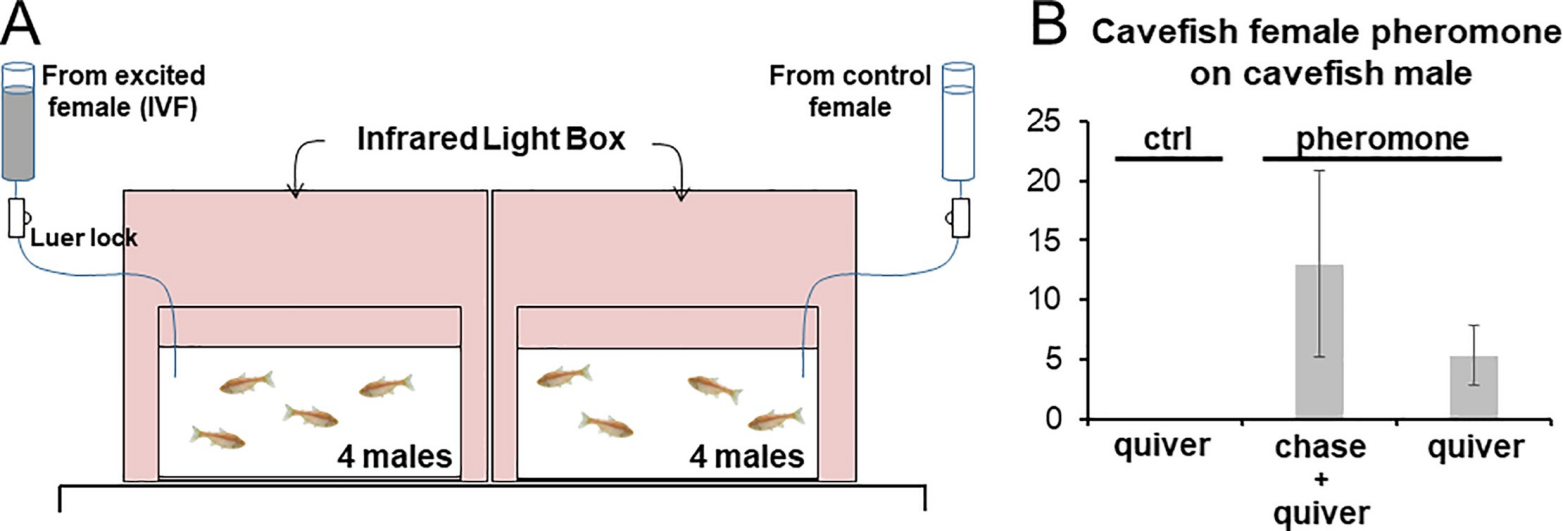
Effect of female pheromonal solution on male reproductive behavior. A, experimental setup. Two tanks with 4 males in each were recorded in parallel. To avoid interference with the perfusion system, the oxygenating pump was removed from the aquaria for this experiment. B, histogram showing the number of quivering, or chasing plus quivering events occurring during 5 minutes between cavefish males upon perfusion of control solution or female cavefish pheromonal solution (n = 3 groups each; mean± SD *per* group).

Solutions enriched in pheromonal substance(s) were obtained from females used for *in vitro* fertilization protocols (See Methods). Two 20 liters’ tanks were prepared, with 4 males in each tank. After 12 hours of habituation, the pheromonal solution or a control solution were perfused during 8 minutes, and infrared videos were recorded (Fig 5A). Three groups of 4 males of each *A*. *mexicanus* morphotype were exposed to pheromonal solutions originating from either surface fish or cavefish females (n = 48 males total were tested).

In the 3 groups of cavefish males exposed to cavefish female pheromonal solution, strong behavioral responses were observed (Fig 5B and S5 Movie). Within 5 minutes, up to 25 events of chasing and quivering were observed between the 4 males in the experimental tank with pheromone perfusion. Importantly, such behavior was never observed in the tank with control perfusion (p = 0.063, U = 0; n = 3 each, Mann Whitney test). This shows that pheromonal signals released by females when they are ready to spawn are able to trigger strong courtship behavioral responses in males.

Surprisingly, in the same assay, cavefish female pheromonal solution had no effect on surface fish males (0 quivering event). Further, surface fish female conditioned water had no effect either, neither on surface fish males nor on cavefish males (0 quivering event). The surface fish female conditioned solution may be ineffective because of the high stress observed in the female during the pheromone collection procedure (contrarily to the cavefish females, see Methods). However, the negative result observed with the cavefish pheromone (which is active since it induces reproductive behaviors on cavefish males) on surface fish males remains unexplained and will be discussed below.

## Discussion

### Courtship behavior

To our knowledge, this is one of the first detailed description of breeding behavior in the laboratory for a characiform, and the first description of breeding habits in a blind cavefish. In the reproductive behavior of the two morphs of the species *A*. *mexicanus*, we could identify 5 very rapid phases: 1) Bottom swimming of the female, 2) Chasing by the males, 3) Quiver swimming of the female with one male, 4) Upward swimming and enfolding of the female by the male while releasing sperm, 5) Twitching and egg-laying by the female. These 5 steps were repeated many times, about once *per* minute, during spawning sessions which lasted about one hour and involved one female and several males.

In all cases, the first sign indicating that spawning was imminent was an increased locomotor activity of the female which attracted the males, suggesting that females trigger reproductive behaviors in males and not the reverse (this does not preclude that males may also release pheromones with primer effects related to reproductive behavior, for example to activate gamete maturation in females [24]). It is probable that such female behavior is elicited when their gametes are mature. Indeed, when attempting to perform *in vitro* fertilization on *A*. *mexicanus*, viable oocytes are obtained only when females are very active, but not just a few minutes before they start increasing their locomotor activity, which suggests that the transition between inactive state and active state with regards to reproduction is very fast (personal observations of laboratory members). This hypothesis is also reinforced by the finding that water where an excited or mature female has been placed is able to elicit chasing and quivering in males (in cavefish). These steps of chasing and quivering appear very similar to the well-described courtship behavior in the model fish *Danio rerio*, a cypriniform [25]. Textbook illustrations of breeding behavior in piranhas [26] or a succinct report of spawning behavior in *Hyphessobrycon eques* [27] (two characiforms) tend to suggest that chasing and quivering are also part of their courtship behavior. An important difference, however, is that the two morphs of *A*. *mexicanus* perform these movements in the dark. Gamete release then occurs in the water column through highly stereotyped and coordinated movements of the male and the female. During this step, the presence of denticles on the male’s anal fin may have a stimulatory role, as previously proposed [14, 15]. The precise enwrapping movement performed by the male around the female’s back, in the dark and in the absence of vision, must rely on other, perhaps mecano-sensory or acoustic, modalities.

Our observations showed that a single female reproduces with several males, in accordance with earlier reports [19]. This confirms the idea that there is no mate choice for surface fish or Pachón cavefish breeding in the dark. We cannot exclude the possibility that in the wild surface fish may also reproduce during the day, in which case the reported female’s preference for large males in lighted conditions would find a potential relevance [18]. Furthermore, the systematic breeding of one female with several males implies that there is a high level of genetic and allele mixing at each generation, an important information for laboratories working on *Astyanax*: in overnight spawns obtained from natural breeding in laboratory tanks, the embryos, juveniles and adults generated which are used for experimentations are genetically diverse.

Inter-morph reproductive behavior was more easily observable in crosses involving female cavefish and male surface fish than the reverse. We interpret this tendency as a consequence of the strong hierarchical dominance and aggressiveness established by surface fish females in groups of fish, and which does not exist in cavefish females, as a corollary of the loss of aggressiveness in Pachón cavefish [28]. This observation suggests that when occurring in the wild, hybridization between the two morphs may be biased towards one direction. Because maternal effects controlling eye degeneration and early developmental events have been described recently in cavefish [29, 30], such a bias towards one direction of cross could be important in terms of the consequences of surface fish introgression into caves on the phenotypes of the progeny.

### Evolutionary aspects

Surface fish and cavefish show the same breeding behavior, and spawning occurs “naturally” with any combination of females and males of the two morphs, without the need of *in vitro* fertilization to obtain hybrid offspring. This suggests that the ability to reproduce in the dark has not been a strong adaptive challenge nor a behavioral bottleneck when surface fish ancestors of the extant cave populations were trapped into caves: surface fish can easily breed in the dark–and they are actually better at finding mates than at finding food in the dark [31, 32]. This also explains the frequent finding of hybrids in some Mexican caves where surface fish are washed during the rainy season. Finally, this fits well with the scenario of recent colonization of caves [4, 5] : the spawning behavior and its trigger have not evolved yet in the cavefish populations after 20–25.000 years (and probably much less generations) spent in their novel environment.

Contrarily to other popular fish models like cichlids or sticklebacks, surface-dwelling *A*. *mexicanus* do not show sophisticated courtships including spawning site selection and preparation of nests, or complex progression of displays involving specific swimming movements, fin erections or changes of color. Their spawning behavior is simple, rapid, and repetitive. And most importantly, it occurs during the night–which goes with a lack of refined visual cues involved in the behavior. Together these features appear like critical “pre-adaptive” traits for effective cave colonization, as already noted by Wilkens [19]. It may also partly explain why only *Astyanax*, among the many fish species inhabiting Mexican rivers, have been able to successfully settle in caves. During our caving field expeditions, we have often witnessed cichlids or poecilids in ponds hosting cave *Astyanax*, and they were always in very poor condition or dying. Cichlids are known for their complex courtship behaviors and their parental care to larvae, which may be incompatible with reproduction in the dark. However, the *Poecilia Mexicana* live bearing species did colonize extreme environments characterized by both sulfur toxicity and permanent darkness. In these fishes the use of non-visual cues has evolved as a novel trait for mate choice in the (eyed) cave population [33], and the strong selective constraints imposed by the double-extreme ecological conditions may have impacted sexual behavior as well.

### Reproduction in numbers

In our experimental conditions of behavioral recording, surface fish females seem to produce more eggs than Pachón cavefish female. It should be noted that these data are based on estimations, as it was impossible to count eggs on all spawning events. In addition, some spawning events may have been missed in cavefish, as their eggs were more difficult to visualize than for surface fish, for unknown reasons which may include the different color and contrast properties of the eggs in the two morphs [34]. As the main factor leading to the finding of a difference between surface fish and cavefish egg production is the proportion of spawning attempts where egg laying was observed, this may constitute an important bias. Moreover, the two morphs were not kept in the exact same conditions in our fish facility (26°C for surface fish; 23°C for cavefish) and the protocol used to induce spawning for movie recordings was also different for the two morphs (decrease to 22°C during 2 days followed by an increase to 26°C for surface fish; progressive increase to 26°C over a week in cavefish). These different procedures might favor a better gametogenesis in surface fish.

However, the difference in egg production estimation in the two morphs is very important: about 3500 eggs *per* female in surface fish, *versus* about 1400 eggs *per* female in cavefish. These estimates are in the order of magnitudes reported for egg numbers produced for the genus *Astyanax* in the literature [16]. One hypothesis to explain the difference in eggs number between the two morphs takes into account the fact that cavefish eggs are bigger than surface fish eggs, with a 35% higher yolk volume in the former [35]. In cave Amblyopsids also, the clutch size is smaller and the egg size is larger than in surface-dwelling Amblyopsids [12], and the same holds true for the cave and sulfur mollies of the species *Poecilia mexicana* [36]. Therefore, these cavefishes may be slowly evolving towards a *K*-strategy favoring the quality of offspring.

There are commonalities in breeding behavior and pattern between *A*. *mexicanus* and the widely-used laboratory fish model, the zebrafish [25]. In both species, spawning sessions for a female lasts 1–2 hours, and courtship is repetitive with many egg-laying events during one such session (12 in zebrafish; 30–60 in *A*. *mexicanus*). However, the number of eggs laid *per* expulsion is smaller in zebrafish (5–20, *versus* about 70 in *A*. *mexicanus*), and the total number of eggs spawned *per* female is about 185 [25]. Overall, the repetitiveness of the spawning behavior in cave and surface *Astyanax* appears like an energetically-costly and labor-intensive activity.

### Chemical communication in *A*. *mexicanus* reproduction

Pheromones are secreted single or mixed odorant molecules that trigger the same social responses in members of the same species. Reproductive behavioral responses to conspecific odors provide the most well studied examples of pheromone function in teleost fish [37, 38]. Perhaps best described in goldfish [39], in many species after ovulation the female releases pheromones of ovarian origin that attract the male and elicits the persistent courtship that accompanies spawning through the stimulation of its olfactory sensory system. Here we have shown that water where a female ready to spawn has been placed is able to elicit chasing and quivering in groups of males, suggesting that the same occurs in *A*. *mexicanus*. The exact nature of the active molecule(s) is unknown, but we can speculate on hormonal pheromones (gonadal steroids, prostaglandins and/or their precursors and metabolites) [38] acting on the olfactory system of the male [19]. Of note, we have tried to elicit courtship behaviors in males with water that had contained eggs freshly squeezed from females (not shown). Such “egg water” does not trigger reproductive behavior (zero quivering event), but rather feeding or foraging behavior, i.e., bottom searching as originally described by Schemmel [40], and in line with the filial cannibalism behavior observed in *A*. *mexicanus* as in many other teleosts [41]. Thus, the ovarian liquid contains distinct molecules which elicit very different behaviors.

Surprisingly, the “pheromone experiment” yielded positive results only with female cavefish substance on cavefish males. While the surface fish female pheromonal solution may be ineffective because of the high stress observed in the female during the pheromone collection procedure (contrarily to the cavefish females), the negative result observed with the active cavefish pheromone on surface fish males is puzzling. Two hypotheses can be drawn. First, the surface fish males may not be able to detect the cavefish pheromone, either because the concentration was too low and they have weaker olfactory skills than cavefish [11, 42] -but note that excellent olfaction capacities have been demonstrated in cavefish only for food-related odors-, or because the odorant molecule(s) released is cavefish-specific and cannot be recognized by surface fish males, which would suggest that this sensory trigger of reproductive behavior has evolved between the two *A*. *mexicanus* morphs. The second possibility is that surface fish males do smell the cavefish pheromone, but this stimulation is not sufficient to trigger chasing and quivering behaviors. Additional stimulation by the female, or reciprocal interactions between the male and the female, for example through acoustic signals in the dark, may be necessary. Indeed, acoustic communication is an important modality in fish reproduction (for examples: [43–45], and it will be interesting to investigate its relevance in the reproductive behavior of cave and surface morphs of *A*. *mexicanus*.

## Supporting information

S1 Movie Reproductive behavior in *A*. *mexicanus* surface fish. The female is indicated by a yellow arrowhead and the male by a blue arrowhead. Egg release is shown by a green arrow. The video should be played at x0.25 speed to see the details.
(MP4)

S2 Movie Reproductive behavior in *A*. *mexicanus* cavefish. The female is indicated by a yellow arrowhead and the male by a blue arrowhead. Egg release is shown by a green arrow. The video should be played at x0.25 speed to see the details.
(MP4)

S3 Movie Reproductive behavior between *A*. *mexicanus* cavefish female and surface fish male. The female is indicated by a yellow arrowhead and the male by a blue arrowhead. Egg release is shown by a green arrow. The video should be played at x0.25 speed to see the details.
(MP4)

S4 Movie Reproductive behavior between *A*. *mexicanus* surface fish female and cavefish male. The female is indicated by a yellow arrowhead and the male by a blue arrowhead. Egg release is shown by a green arrow. The video should be played at x0.25 speed to see the details.
(MP4)

S5 Movie Quivering behavior between cavefish males upon cavefish female pheromone perfusion. The left tank is perfused with control water solution and the right tank is perfused with female pheromone. Quivering between males is observed in the later.
(MP4)
